# The peer review process for awarding funds to international science research consortia: a qualitative developmental evaluation

**DOI:** 10.12688/f1000research.12496.3

**Published:** 2018-01-16

**Authors:** Stefanie Gregorius, Laura Dean, Donald C Cole, Imelda Bates

**Affiliations:** 1 Capacity Research Unit, Liverpool School of Tropical Medicine, Pembroke Place, Liverpool , L3 5QA, UK; 2 Dalla Lana School of Public Health, University of Toronto, 155 College St. , Toronto, ON , M5T3M7 , Canada

**Keywords:** Peer review, review panels, grant applications, research consortia, research capacity, Africa

## Abstract

**Background: **Evaluating applications for multi-national, multi-disciplinary, dual-purpose research consortia is highly complex. There has been little research on the peer review process for evaluating grant applications and almost none on how applications for multi-national consortia are reviewed. Overseas development investments are increasingly being channelled into international science consortia to generate high-quality research while simultaneously strengthening multi-disciplinary research capacity. We need a better understanding of how such decisions are made and their effectiveness.

**Methods: **An award-making institution planned to fund 10 UK-Africa research consortia. Over two annual rounds, 34 out of 78 eligible applications were shortlisted and reviewed by at least five external reviewers before final selections were made by a face-to-face panel. We used an innovative approach involving structured, overt observations of award-making panel meetings and semi-structured interviews with panel members to explore how assessment criteria concerning research quality and capacity strengthening were applied during the peer review process. Data were coded and analysed using pre-designed matrices which incorporated categories relating to the assessment criteria.

**Results: **In general the process was rigorous and well-managed. However, lack of clarity about differential weighting of criteria and variations in the panel’s understanding of research capacity strengthening resulted in some inconsistencies in use of the assessment criteria. Using the same panel for both rounds had advantages, in that during the second round consensus was achieved more quickly and the panel had increased focus on development aspects.

**Conclusion: **Grant assessment panels for such complex research applications need to have topic- and context-specific expertise. They must also understand research capacity issues and have a flexible but equitable and transparent approach. This study has developed and tested an approach for evaluating the operation of such panels and has generated lessons that can promote coherence and transparency among grant-makers and ultimately make the award-making process more effective.

## Introduction

The use of peer review by expert panels is a well established method for assessing scientific research and for evaluating grant applications ( Abdoul *et al.*, 2012; Coryn *et al.*, 2007; Lawrenz *et al.*, 2012; Wooten *et al.*, 2014). Most of the literature on peer review focusses on how to ensure transparency and reliability in editorial peer review. However, except for Lamont’s (2009) in-depth work, a Cochrane review ( Demicheli & Di Pietrantonj, 2007) and a recent review ( Guthrie *et al.*, 2017), little research has been conducted into the peer review process of grant applications; the few studies that have been conducted focused on individual research projects ( Abdoul *et al.*, 2012) and national research collaborations ( Klein & Olbrecht, 2011).

Overseas aid investments in science and technology are increasingly being channelled into international consortia models ( El Ansari *et al.*, 2007; UKCDS, 2015). Such research consortia usually comprise research institutions in high-income countries (HIC) and low- and middle-income countries (LMIC). Such consortia generally aim to generate innovative science through world class research and to strengthen research capacity at the individual, institutional and national/international level. For the HIC country partners, these collaborations provide a rich experience and understanding of working in developing countries and opportunities to adapt innovations for different contexts. The LMIC institutions benefit from exposure to a diversity of world class equipment and facilities that help strengthen individual and institutional research capacity ( Dean *et al.*, 2015; Syed *et al.*, 2012).

Much of the literature on peer review has focussed on review of articles for publication. Peer review by panels evaluating research applications has received much less attention ( Klein & Olbrecht, 2011) and we could find no publications about the peer review process for multi-national science consortia. There is therefore almost no evidence to guide good practice for peer review of these complex applications. In order to select consortia for funding, peer review panels have to assess consortia’s potential for achieving the dual aims of generating high quality science and strengthening LMIC research capacity at all three levels (i.e. individual, institutional, societal). These aims are complex and interlinked, and so the review panel need to have topic- and context-specific expertise and a flexible but equitable approach ( Wessely, 1998). Given the amount of money invested globally in trans-national research consortia, there is a pressing need to understand how funding decisions are made and to develop an evidence base that can help to promote coherence and transparency within and among grant-makers, to ultimately make the process more effective.

This article describes the use of qualitative research to explore the peer review process used for awarding grants to ten multi-national natural science research consortia. The scheme was specifically designed and budgeted to support ten consortia which were funded through a UK grant-making institution over two annual rounds of applications (2014 and 2015). Each consortium consists of three research institutions in sub-Saharan Africa (SSA) and one in the United Kingdom (UK). Applications were restricted to three research priority areas: renewable energy, soil-related science and water and sanitation. In addition to generating high-quality research, a key goal of the programme was to strengthen the research capacity of universities and research institutions in SSA by strengthening research infrastructure and the development of sustainable research networks. This was to be achieved by establishing multidisciplinary partnerships between the UK and SSA, strengthening research training capacity in SSA and establishing a PhD scholarship scheme with shared UK-SSA supervision.

The study into the peer review process was conducted by the Capacity Research Unit (CRU) at the Liverpool School of Tropical Medicine (LSTM). CRU is an independent research team, external to the grant-making body. CRU’s role was to undertake a developmental evaluation of the programme, conduct research into the research capacity strengthening processes of the programme and generate learning that could be used to improve the programme in (near to) real-time. Developmental evaluation ( Patton, 2011) is an evaluation approach that helps to introduce change within uncertain and complex environments. It provides feedback to programme staff to inform a quality improvement loop. Consequently, this paper describes the process for selecting consortia in round one and using learning from this round to adjust the round two selection process. The research focused on exploring how closely the peer review selection process matched the overall goal of the programme, how reviewers used the assessment criteria and how final funding decisions were reached.

## Methods

In the absence of validated, published methods for research in evaluating peer review of multi-national research consortia, we applied relevant, well-established, qualitative research methods to collect data at three stages of the selection process: pre-award document review, observation of the award-making panel meetings and post-award interviews with panel members. The selection panel consisted of 20 peer reviewers. The face to face meetings of the panel were attended in person by 17 (round one) and 16 (round two) reviewers. Panel members unable to attend the face to face meeting provided written comments. The panel comprised three women and 14 men (round one) and three women and 13 men (round two). Three reviewers for each round were from Africa and the rest were from the UK or European Union. Their expertise covered water and sanitation (two per round), soil science (two per round), renewable energy (seven per round) and other relevant disciplines (5–6 per round).

Structured overt observations of the two panel meetings were carried out to systematically document the process ( Supplementary file 1 and Supplementary file 2) and semi-structured interviews ( Supplementary file 3) were conducted with purposively selected panel members after the meetings. The interviews were designed to gain in-depth understanding of how the assessment criteria were understood and used and perceptions about the overall selection process. They were also used to validate data obtained from observations of the selection panel meetings.

### Document reviews

Among the 73 eligible applications (26 in round one, 47 in round two) submitted to the award-making institution, 34 were shortlisted for full review (15 in round one, 19 in round two): 6/6 (round one/round two) in renewable energy, 4/8 in soil-related science, and 5/5 in water and sanitation. Each eligible application was scored by three panel members who were assigned applications on the basis of their relevant expertise. Applications that met the assessment criteria and had highest scores were shortlisted. Each shortlisted application was reviewed by at least five external specialists and their comments taken into consideration during the final selection meeting.

Guidance notes for panel members on the conduct of the face to face meeting and the assessment criteria, and summary details of the shortlisted applications for both rounds, were provided to us by the grant-awarding institution. We were not involved in the shortlisting process. We used a pre-designed matrix to extract information from the panel guidance notes about the assessment criteria against which applications were to be judged, the role of the panel members, the role of the panel chair and the panel code of conduct.

### Observation of peer review selection panel meetings

Overt observation of the panel meetings involved the direct observation ( Lamont, 2009; McNaughton *et al.*, 2013) of the panel in their natural setting; as observer-researchers we did not participate in the process. The observations of each panel review meeting were conducted by three (round one) or two (round two) researchers. The researchers gave a brief presentation at the beginning of each meeting outlining the role of CRU within the programme and the purpose of their observations during the panel meetings. Panel members were given the opportunity to ask questions and the researchers gave an undertaking to maintain confidentiality and anonymity.

An iterative approach was used for the observation of the round one panel meeting to allow flexibility in the research approach. This was important as there was no published relevant research in this area and there were no appropriate data collection tools or findings that could be used or adapted. We used the assessment criteria in the panel guidance document to develop tools that enabled data to be collected against each criterion during observation of the selection panel meeting (see box 1 and box 3). In addition, the data collection for the observation of the panel meeting for the first round of shortlisted applications was partially influenced by a Swedish Research Council publication that broadly explored the *modus operandi* of its evaluation panels ( Ahlquist *et al.*, 2013). This document primarily influenced the development and content of observation matrices used for note-taking during the overt observation.

Box 1. **Selection criteria presented at the start of the peer review panel meeting**

***Primary considerations***

- Quality of the research
- Quality of the research teams involved
- Quality of candidates for PhD scholarships (if already identified)
- Quality of the training/supervision plans for:
  - PhD Students covered by scholarships
  - Wider training programme for other researchers and technicians

***Additional considerations:***

- Indication of host institutions’ capability and willingness to utilise the programme for the development of their own research strategy
- Diversity: good spread across the SSA region, including non-Anglophone countries
- Spread across the three priority research areas
- Support for early to mid-career scientists
- Support for female scientists

**Box 2.** Summary of findings from round one and actions taken by grant making organisation

Findings from round one	Recommendations for changes	Actions taken by grant makers for round two
Some inconsistencies in assessment criteria with little explanation on importance and weighting	Panel guidance notes for round two should include more details about the assessment criteria, including weightings	Clarity and specificity of assessment criteria guidelines were improved (see box 3 for details)
The focus and order of discussion was slightly different for each application	Develop a more standardised approach/guidance to ensure that all assessment criteria and their differential weightings are discussed adequately and in order. A possible approach could be: - all priority assessment criteria presented by main and secondary speakers - any other comments not covered in the assessment criteria by main and secondary speakers - consultation of all panel members until decision is reached	Clarity and specificity of assessment criteria guidelines were improved (see box 3 for details)
Most of the panel members were UK-based, although the programme goal is to strengthen science research capacity in sub-Saharan Africa	Diversify panel composition to include more panel members from SSA and non-Anglophone backgrounds	Panel members were the same for both rounds although there were some differences in availability and attendance between the two rounds.

Box 3. **Assessment criteria provided for panel members for round two (revised based on experiences from round one)**

1. Applications should initially be assessed based on scientific strengths by considering the following points:
  • Excellent quality of overall research programme
  • Strong individual research projects
  • Strong research background of scientists
  • Strong track record of host institutions
  • Excellent quality of proposed PhD research projects (including how well they integrate with overall research project)
  • Financial plan
2. Once the scientific strength of the application has been determined then the strength of capacity strengthening should be assessed considering the following points:
  • Host institutions have capability and resolve to utilise the programme for the long-term development of their own research strategy/aspiration
  • Wider training programme for other researchers and laboratory technicians
  • Clearly research active lead members
  • Likelihood that support will lead to institutional changes and long-term benefits for the African partners
3. Additional points to consider:
  • Consortium lead members who are female
  • Africa lead members that are early to mid-career scientists
  • Multi-disciplinary applications
  • Inclusion of non-Anglophone countries

For the round one panel meeting, two separate matrices were used to capture data on a) the contents of each application and b) panel members’ contributions and interactions ( Supplementary file 1). The matrices ensured data were captured for assessment criteria areas of scientific excellence, research capacity strengthening and additional criteria (e.g. gender, career stage). It also enabled information to be captured on the selection process and panel members’ contributions (e.g. the process and format used to discuss each application, time spent on various aspects of each application including applicants’ demographic and professional backgrounds, details of how and which assessment criteria were used and discussed and other communication among the panel members).

For the round two panel meeting, the observation matrices were revised based on lessons learned from observation of the round one meeting. The main changes were to combine the two matrices into one with sub-divisions for key assessment themes (scientific strength, research capacity strengthening, other assessment criteria, themes not covered in assessment criteria). This matrix was completed for each shortlisted application as it was discussed by the panel members ( Supplementary file 2). The observation of the round two panel meeting used the same researcher’s observation guide as in round one to ensure methodological consistency. As in round one, the round two observation focussed on processes and contents, verbal and non-verbal communication of panel members and any other relevant observations, with particular emphasis on which and how assessment criteria were used. This was because the award-making institution had made adjustments to the assessment criteria - to improve clarity and to make it compulsory to include strengthening of laboratories - based on our recommendations after the first panel meeting.

***Data management and analysis of panel meeting observations.*** All the researchers’ hand-written observation notes were transferred onto an Excel spreadsheet. These data were coded and analysed under the *a priori* categories of ‘assessment criteria’ and ‘other observations’. Items with the same code that emerged from the data were amalgamated into themes during debriefing discussions among the researchers after each panel meeting observation. This process facilitated a balanced interpretation of the data and helped reduce the subjectivity of the researchers’ interpretations. The frequency with which key items were discussed at the meeting was also calculated. The datasets concerning the observations of the panel meetings have not been made available because they contain sensitive information and it was not possible to anonymise them without losing the meaning of the data.

### Interviews with panel members

Semi-structured interviews ( Supplementary file 3) with nine (six in round one, three in round two) purposively selected panel members were conducted after each of the two meetings to explore their views and perceptions of the award-making process. The choice of panel members to interview was designed to maximise diversity in nationality, expertise profile and gender. Interviews were conducted by two researchers by phone/Skype within three months of the panel meeting.

The assessment criteria were also used to inform the guides used to interview panel members. The interview guide topics included panel members’ experiences and perceptions of the award selection process, their role in the panel and their understanding of scientific excellence, research capacity strengthening and research partnerships. Since these interviews revealed some differences between interviewees’ responses, the interview guide was revised after round one so that these differences could be explored in more depth during the round two interviews. Interviews lasted 30–60 minutes. They were audio-recorded (with permission) and the interviewers took notes summarising the main issues discussed under each topic ( Dataset 1). Interviewees were assured that the information they gave would be treated confidentially and anonymously to avoid the possibility that panel members’ quotes could be attributable. Since the number of interviewees was small, demographic details for interviewees’ quotes have not been provided in order to maintain anonymity.

Notes of observations from round 1 and round 2 panel meetings Click here for additional data file. Copyright: © 2018 Gregorius S et al. 2018 Data associated with the article are available under the terms of the Creative Commons Zero "No rights reserved" data waiver (CC0 1.0 Public domain dedication).

***Data management and analysis of interviews.*** All hand-written notes were transcribed electronically and checked against the recordings for accuracy. The data in the notes were then coded using codes which were developed iteratively based on themes that emerged from the transcripts and agreed among the research team. Once coding had been completed, links within and between codes were explored and data were grouped into higher level themes to allow for data interpretation and explanation. The panel guidance notes were also used in the analysis to determine how they were understood and followed by panel members during the meetings.

### Ethical considerations

The study was approved by the Liverpool School of Tropical Medicine Research Ethics Committee (ref Research Protocol 13.14RS)

## Findings

In line with the developmental nature of this study, the findings are presented as a narrative, following the chronological order of the events covering the pre-award (document review), award-making (observation of panel meetings) and post-award (interviews with panel members) stages.

### Round one

***Pre-award process.*** The award-making institution received 26 eligible applications for round one of the programme grant. During the initial assessment stage, each panel member was allocated a selection of the eligible applications based on their area of expertise. Each application was assessed independently by three panel members using scores between one (poor) and seven (outstanding) against the assessment criteria. Fifteen applications were shortlisted for external review, six in water and sanitation, six in renewable energy and three in soil-related science. At least five external specialists assessed each application and their reports were sent to the panel members prior to the panel meeting. Their scores were collated and applications were ranked according to their average score. Each application, with its external specialists’ reports, was allocated to three of the panel members to lead on the discussion during the award-making meeting.

***Award-making panel meeting.*** The round one panel meeting started with a general introduction of all participants, followed by a presentation by the award making institution giving relevant background information about the programme and a brief outline of the selection criteria to be used when discussing the applications during the meeting ( box 1).

These assessment criteria reflected those in the panel guidance notes. However, an indication of the differential weighting between ‘primary’ and ‘additional considerations’ was provided in the introductory presentation at the panel meeting but was not included in the written guidance the panel members received in advance of the meeting.

*Process:* The overall format of discussion of each application was consistent throughout the meeting and the applications were not discussed in any particular order (e.g. alphabetical) which may have introduced bias. One panel member led on each proposal and presented their opinion of the application before the other two panel members contributed their comments. After the discussion of the application by the three panel members assigned to that application, all panel members were invited to express their views about the proposed project.

Applications were assigned to each panel member according to their area of expertise while aiming to also provide diversity in terms of Francophone or Anglophone and UK or SSA-based. The observation revealed that the three panel members assigned to discuss a selected application demonstrated a thorough understanding of what was written in the proposals and took into account the applicants’ expertise and backgrounds. Panel members consistently made use of the external reviewers’ comments to inform discussions about the applications. A summary report of the discussions was provided for successful applicants.

In total panel members spent 189 minutes (excluding breaks and general discussions) discussing the 15 applications (average 13 minutes per application), spending an average of 15 minutes on applications they recommended for a grant and 11 minutes on proposals that were not successful. In one instance, panel members spent 20 minutes on a ‘maybe’ application that straddled the boundary between success and rejection. It was decided that ‘maybe’ applications would be discussed further after the panel members had gone through all proposals. The rationale was that this allowed for more informed decisions of ‘maybe’ applications as panel members could compare these to all other applications.

*Content:* The observations revealed substantial variations in how each application was presented and therefore how it was discussed by the panel members. Panel members were not asked to use a specific format for presenting applications and, although most spoke to several of the assessment criteria, not all criteria were discussed for each application (see Table 1 for frequency of themes discussed for each round). Scientific excellence and innovation were given the greatest attention by panel members as well as the applicants’ credentials (e.g. number of publications, journal impact factors, frequency of publication) and success in obtaining grants, including prior collaborative projects.

**Table 1.** Themes discussed and their frequencies during each panel meeting.

		Number of times mentioned during meeting		
Themes	Integration of themes	Round 1	Round 2	Overall
Scientific strengths				
	Relevant skills & work experience, including background-track record of PI and co investigators)	12	10	**22**
	Scientific excellence	15		**15**
	Selection/composition of partners - Quality of researchers and teams in research priority area	7	6	**13**
	Novelty/innovation of research		11	**11**
	Quality of research methods used		8	**8**
	Financial plan including equipment	5	2	**7**
	Collaborative experience/connection of team members	6	1	**7**
	Publication record of scientists		7	**7**
	Research leadership of scientists		5	**5**
	Quality of scientific hypothesis/objectives/research questions		5	**5**
	Africa experience of UK scientists	1	1	**2**
	***Total***			***102***
Strength of capacity strengthening plans				
	Development relevance	5	7	**12**
	PhD plan and support	5	2	**7**
	Specific training of/for: - use of equipment - technicians - research specific training - PhD student supervision - career progression possibilities - budget for capacity strengthening		each training mentioned once	**6**
	Research capacity strengthening	5		**5**
	Research capacity training plan	4		**4**
	Likelihood for institutional change through South-to- South learning		4	**4**
	Sustainability aspect	2		**2**
	Existing research infrastructure African institutions		2	**2**
	Relevance of research training		2	**2**
	Strengths of UK institution with regard to capacity strengthening		1	**1**
	Including postdocs in programmes for sustainable research capacity strengthening		1	**1**
	***Total***			***27***
Other assessment criteria				
	Gender issues considered	6		**6**
	Anglophone/francophone balance	3		**3**
	Career age of African scientists		3	**3**
	Multidisciplinary application of research project		3	**3**
	Female lead members		1	**1**
	***Total***			***16***
Themes not covered in assessment criteria				
	Explicit reference to reviewers’ comments and scoring/Reviewers’ scoring	4	11	**15**
	Integration of research projects		7	**7**
	Dissemination/communication plans	2	3	**5**
	Feasibility of project	4		**4**
	Clarity of proposal	4		**4**
	Writing style and structure	4		**4**
	Transferability of results		2	**2**
	Risk assessment		2	**2**
	Dissemination to other institutions	1		**1**
	Research capacity needs assessment	1		**1**
	Information on research progress	1		**1**
	Additional funding	1		**1**
	Comparison to other proposals	1		**1**
	***Total***			***48***

Research capacity strengthening was discussed for almost all applications but for a shorter time and in less detail than scientific excellence. It was often used as an additional selection criterion to differentiate between two applications of similar quality. The observation revealed that panel members commonly used the term ‘capacity strengthening’ in relation to research training for individuals, including PhD candidates. There were limited discussions surrounding broader aspects of capacity strengthening such as PhD supervision plans and the institutional research infrastructure. Other aspects of some, but not all, applications were discussed by the panel, such as presentation of the application, budgets and applicant’s career age (i.e. research-active years since their PhD) especially in relation to their potential (in)experience of managing large awards. Unsuccessful applicants received feedback based on a summary of the discussions at the panel meeting and pre-meeting written reviews.

### Post award interviews with panel members

Interviews with panel members after the selection meeting showed that they were positive about the process and emphasised the high levels of rigour, collaboration and balanced expertise ** of the panel members. Their assessment was ** primarily based on their previous experiences of serving on other review panels. Responses to questions about how the panel worked as a team showed that all those interviewed agreed that the committee ** was ** cooperative and respectful and that panel members were given enough time to speak ** and listened to each other. Interviewees highlighted that for each application consensus was reached based on thorough and constructive ** discussions. A few interviewees attributed the successful functioning of the panel to effective chairing of the meeting.

Specific expertise in one or more of the three priority research areas of the programme was quoted as the main reason why panel members believed they were approached to participate in the review process. One panel member also thought she had been selected because she was female and an African panel member felt he was invited because he could provide an African perspective and contextual expertise. He noted that during the review process he ‘ *scrutinised the applications with regard to development relevance for Africa’.*

Most interviewees felt that they were provided with clear guidelines about how to review the applications. However, interview data mirrored the researchers’ observations that for some aspects panel members had different understandings about the interpretation or weighting of the assessment criteria, particularly relating to the balance between scientific excellence and research capacity strengthening, as the following two quotes exemplify:

*‘… the starting point was quality of science … the tiebreaker was actually the capacity building aspect. Maybe naïvely I thought it would be the other way round … So I was perhaps surprised that the quality of science dominated …’ (Interviewee 5)*
*‘It could have been the best science ever, but if it did not have the capacity building, it wasn’t going to get funded. I did feel genuinely that we were steered to look at that and give that significant weighting.’ (Interviewee 3)*

Interview data showed that panel members had a common understanding of scientific excellence, focussing primarily on innovative research projects and the applicants’ publication and grant records. However, responses to interview questions about what they thought were key characteristics of research capacity strengthening showed a range of different responses. Characteristics mentioned by the various panel members included: sustainable physical and human infrastructure of institutions, multi-disciplinary and international collaborations, training of researchers at different levels (including students, postdoctoral researchers and supervisors), research uptake strategies and sustainable monitoring and evaluation processes. There did not appear to be a common understanding of the concept of ‘research capacity strengthening’ among the panel members.

***Learning generated by developmental evaluation of round one selection process and used to influence round two.*** Recommendations for improvements to the selection panel process for round two were derived from the findings from research into round one selection processes ( box 2). These recommendations included improving the clarity and specificity of assessment criteria guidelines, a more standardised structure for presenting each application and increasing the diversity of the panel.

### Round two

***Pre-award process.*** The shortlisting process for the 47 eligible applications was the same as for round one except for the use of revised guidance on assessment criteria (see box 3).

***Award-making panel meeting.*** The overall format of the panel meeting was similar to round one with presentation of the applications by the relevant lead panel members, followed by open discussion by the whole panel.

*Process.* Compared to round one, there was less variation in how the applications were discussed and more consensus in the decision-making. In total panel members spent 178 minutes (excluding breaks and general discussions) discussing the shortlisted 19 applications (average 9 minutes/application versus 13 minutes/application in round one). The relative time spent on successful, unsuccessful and ‘maybe’ applications was similar to that in round one. Panel members spent on average 11 minutes on the 5 applications they recommended for a grant (15 minutes in round one), 9 minutes on the 14 unsuccessful proposals (11 minutes in round one) and 14 minutes on the ‘maybe’ applications (20 minutes in round one).

*Content.* Compared to round one there was less variation in the focal areas of the discussions concerning scientific strength. These areas covered reviewers’ scoring, the novelty of the research, research methods and how well individual research projects were integrated within a consortium (see Table 1 for frequency of themes discussed for each round). The panel also discussed areas not covered in round one and not included in the revised assessment guidance, such as dissemination of research findings, transferability of results and risk assessments. Compared to round one there was also more discussion on development relevance for Africa and greater consensus among the panel regarding applications recommended and not recommended for funding which was reflected in the shorter time needed to discuss each application in round two compared to round one.

There were no major differences between round one and round two in how panel members discussed the credentials of scientists and their complementarity within consortia. Discussions focused on applicants’ complementary research backgrounds, especially their publication and grant records, frequently using the term ‘well-published’.

Research capacity strengthening components of applications were discussed in 13 out of the 19 applications; these were not necessarily those in which scientific strength was considered to be possibly insufficient. The panel considered many of the points under research capacity strengthening that were listed in the revised guidance notes (e.g. relevance of training, equipment and statistics training, cross partner training). However, the training of laboratory technicians was only discussed once and there was limited consideration of how proposals would impact on the wider research infrastructure. Although sustainable research networks were mentioned as a goal of the scheme this topic was not explicitly discussed by the panel members.

### Post award interviews with panel members

All three interviewees assessed the award-making process positively, commenting particularly on how well the panel worked as a group, the chairing of the meeting and how the panel made informed decisions about funding. They highlighted a high level of mutual respect among the panel members and an increased level of familiarity with the process based on experiences from round one. Despite using the revised assessment guidance notes in round two, the interviews revealed that there was still some ambiguity in the interpretation of the weighting of the assessment criteria. Whereas two panel members highlighted the importance of scientific strength, noting ‘ *we have to select science, capacity strengthening was always the second tier*’, another panel member (Interviewee 7) explained:

*‘When I review them [applications], I am likely to look at capacity enhancement first, rather than the science, and there is a little bit too much focus on science, and not on the capacity aspect or training aspect, the training programme.’*

One panel member felt that capacity strengthening was given less attention during this panel meeting compared to the previous one, but in contrast another interviewee (Interviewee 8) noted that there was more consensus around the focus on capacity strengthening issues:

*‘I think that [discussion of capacity strengthening] is one thing that I would say was stronger, the 2015 round compared to the 2014…..I think the panel really did appreciate and did really grasp some of the key issues in capacity strengthening this time to a significantly greater extent than during the previous occasion.’*

In line with the panel guidance notes, interviewees noted that additional points, such as gender and the career level (i.e. early- or mid-career) of African scientists and non-Anglophone applications, were discussed in less detail than scientific strength and capacity strengthening.

## Discussion

This study primarily used structured, overt observations and semi-structured interviews to explore the peer review process used by an award-making institution to select ten UK-Africa natural science research consortia over two annual rounds. The selection panel had been constituted to include expertise across the three different scientific areas (i.e. renewable energy, soil-related sciences and water and sanitation), women and Francophone and African members. The panel constitution was generally consistent for both rounds of awards which facilitated inter-round comparisons and helped ensure consistency in standards between the two rounds. Using the same panel for both rounds had advantages in that during the second round they achieved consensus more quickly and increased their focus on development aspects in addition to scientific aspects. Consideration was given to increasing the diversity of the panel for round two, but, ironically given the funding criterion of ‘support for women scientists’, this proved difficult given the relative paucity of women and African scientists in these three research areas. It was this capacity problem that the programme was designed to address. Members of funding panels can benefit directly from the experience ( Guthrie *et al.*, 2017) because it expands their knowledge of the research process, so not including more women on the panel may perpetuate the paucity of women scientists. Funding and grant-making agencies have a key role to play in ensuring diversity on peer-review panels ( Lamont & Huutoniemi, 2012)..

The shortlisting process was similar for both rounds of awards and broadly followed the benchmark process used by many grant-awarding bodies ( RCUK, 2006). The benchmark describes how submitted proposals should be assessed by some combination of external referees, peer review panels and expert programme managers and final reports should be provided for successful applications. For this UK-Africa award, the final assessment panel comprised only scientists. Each eligible application was scored by three panel members. Those with the highest scores that were considered to have met the assessment criteria were shortlisted. Each shortlisted application was reviewed by at least five external reviewers and their reviews were considered alongside the panel members’ reviews in the final selection meeting. Scores for each application were pooled across panel members and then each application was discussed individually. Notwithstanding this in-depth process for making decisions about applications, there is evidence that panel discussions may not improve the reliability of such evaluations ( Fogelholm *et al.*, 2012; Jayasinghe *et al.*, 2006).

The study showed that panel members demonstrated a good understanding of the contents of the applications and took the relevance of applicants’ credentials into consideration in their decision making. Panel members themselves were positive about the process, particularly the rigour, collaboration and diversity of expertise. Compared to their experience on other review panels, some members noted that the task for this panel was particularly challenging because of the need to cover three different natural science areas and many different countries. Compared to round one, in round two there was more discussion on development relevance for Africa, suggesting increased awareness of its importance in meeting the goals of the programme. There was also greater consensus among the panel regarding applications to be recommended or not recommended for an award. The relative time spent on different types of applications was consistent between the two rounds. The shortest time was spent on unsuccessful applications and the longest time on those initially classified as ‘maybe’. This timing pattern has been observed elsewhere ( Ahlquist *et al.*, 2013) and suggests that negotiations about funding decisions are most critical for applications that are close to the boundary.

In general, the panel based their assessments of the applications on the guidance notes. However, even for round two there were some inconsistencies in the written and oral information provided to the panel members concerning differential weighting between primary and secondary considerations. In response to findings from the evaluation of the first round of award selection, the clarity and specificity of assessment criteria guidelines were improved. The subjectivity and lack of consistency in the use of criteria by reviewers can adversely affect the reliability and validity of the assessment. Since this may result in mistrust among grant applicants about the review process it has been found that it is important to ensure transparency in the review process by, for example providing definitions for each assessment criterion ( Abdoul *et al.*, 2012). A statistical model has been proposed to analyse peer review scores for grant applications which accounts for differences in reviewers scoring patterns, ratings from preliminary scores and group discussions and the final results. Initial findings from application of the model to a US peer review system suggested that it would have resulted in a 25% change in the funded proposals ( Johnson, 2008). It is also recognised that the cognitive and professional perspectives of reviewers influence their decisions, so peer review cannot be an objective process characterised by a consistent application of a set of criteria ( Lamont & Huutoniemi, 2012). The need for consistent criteria seems to be of more concern to science disciplines than social science and humanities, perhaps because the latter disciplines are more conscious of the effect of intersubjectivity ( Lamont & Huutoniemi, 2012).

Panel members were not given a specific format for presenting their summary reviews of applications. This resulted in significant variations in the sequencing and coverage of topics discussed for each application. Although scientific excellence and the applicants’ credentials were always discussed, some assessment criteria were not used at all for some applications. This may have been appropriate since, if the main criteria of high quality science and suitable applicants were fulfilled, then other factors may not have warranted consideration. However, it did mean that for a few applications, secondary criteria such as research capacity strengthening, gender and career stage, were not discussed. If the award-making institution wants such factors to be taken into account for every application in an unbiased non-subjective way, then it may be necessary to introduce a scoring system, with clear definitions for each score, as part of the panel meeting process. There was much emphasis throughout the review process on scientific excellence. However the notion of ‘excellence’ in scientific research has been criticised as having little meaning and it has been suggested that it should be replaced by ‘soundness and capacity-building’ ( Moore *et al.*, 2017). Feedback was provided to unsuccessful applicants summarizing strengths and weaknesses of the proposal. There is scope to improve the amount and quality of such feedback given the amount of time and effort that goes into reviewing each proposal. Such feedback can be very helpful for applicants ( Barnett *et al.*, 2015) and grant-making bodies may want to consider how they could improve the quality of feedback for example, by providing audio transcripts of panel meetings.

There were significant variations in the panel members’ understanding of the term ‘research capacity strengthening’, which mirrors the current lack of clarity regarding the definition of this term in the literature ( Dean *et al.*, 2017; Gadsby, 2011). When the UK-Africa programme was first conceived, the capacity strengthening element focused on training for individuals. However since then the importance of the research environment in enabling researchers to utilise their training effectively has been increasingly recognised. This has resulted in a shift in research funders’ focus towards strengthening institutions’ research systems and infrastructure ( Bates *et al.*, 2014; ESSENCE, 2014). To facilitate a common understanding of the concept of ‘research capacity strengthening’ the panel would have benefitted from clearer guidance about the definition as applied to this programme and more detailed assessment criteria. In evaluating complex applications that address the dual goals of high quality science and research capacity strengthening there is a tension between having enough assessment criteria to be able to evaluate contextual and collaborative variables without over-burdening the reviewers and making the assessment process too cumbersome. For panels that have to assess these dual goals there is also a balance to be struck between traditional scientific criteria and criteria related to, for example, research culture and infrastructure, that are important to achieve training and capacity building objectives ( Wooten *et al.*, 2014).

### Study strengths and limitations

The overt observation method was chosen because it would yield a rich amount of data when exploring the award-making process, how panel members used the guidance notes and how the two rounds of award-making differed from each other. The presence of more than one researcher taking independent notes at each meeting and discussing findings among researchers immediately after the meeting promoted an unbiased interpretation of the data and enabled the researchers to document processes and interactions that the panel members themselves might consider self-evident or not worth mentioning. However, overt observation does not allow exploration of the cause of observed phenomena and it is not possible to be confident that observations represent normal behaviours since subjects may act differently when they know they are being watched ( McNaughton *et al.*, 2013). To mitigate this possibility the observation data were compared with information from the semi-structured interviews with panel members. The interviews provided information about experiences of the panel members which could not be captured through observations. It is possible that interviewees might have given responses that did not truly reflect their feelings as they were aware that information might be shared (anonymously) with the award making institution. Therefore, before each interview the interviewees were reassured that their information would be treated confidentially and anonymously.

### Generalisable lessons learnt about evaluating complex multi-national research consortia applications

1.    The constitution of the panel should be diverse enough to reflect the scientific topics, LMIC context, gender and language of the applications and should be maintained across different rounds of awards to ensure consistency.

2.    The shortlisting and selection process, with involvement of external reviewers, pooled scores and discussions during the panel meeting, is in line with funders’ benchmarks and can be applied to these complex consortia applications to provide a rigorous and equitable selection mechanism.

3.    Assessment criteria need to be defined in terms of content and their weighting. The number of assessment criteria should be limited to those most revelvant to the programme’s aims to enhance consistency during the discussions.

4.    The guidelines should specify whether criteria such as gender and career stage must be considered in all cases, or whether they should only be used as ‘tie breakers’ and how much weight they should carry.

5.    For programmes which have dual (but not exclusive) aims of generating high quality research and strengthening research capacity:

a.    the relative weighting of each of the goals needs to be made explicit
b.    assessment guidelines need to be clear about how to evaluate the research capacity strengthening component of applications and panel members need to all have the same understanding of what research capacity strengthening means in the context of the programme.

## Conclusions

This study provides an evaluation of the complex process of awarding grants to multi-national, multi-disciplinary science research consortia. It provides insights into how the award process was designed and conducted and gauged how closely the process matched the overall aims of the programme which were to support both high quality science research and development of research capacity in the African institutions. Research consortia are one of the most popular ways of funding collaborative multi-national research because, compared to a single site study, they provide additional benefits such as greater generalisability of findings and a more comprehensive understanding of the issues. Multi-disciplinary consortia can also create synergies that make them more influential in catalysing changes in policies and programmes and they can help to address inequality of resources and research opportunities among partners. The diversity and complexity of multi-partner consortia presents challenges and potential risks, such as inequity between partners and lack of cohesion around common goals and expectations ( Dean *et al.*, 2015), that need to be considered in the selection process. It is important for award-making institutions involved in these complex, consortia-based research models to put in place mechanisms for robust and systematic learning and to be flexible enough to incorporate changes into subsequent selection processes to make the award-making process more effective. All those involved in grant-making need to acknowledge and analyse the uncertainty in the peer-review process, to recognize that there is a lack of evidence and openness around the process, and to promote experimentation into ways of funding research that are efficient and transparent ( Guthrie *et al.*, 2017)

## Data availability

The data referenced by this article are under copyright with the following copyright statement: Copyright: © 2018 Gregorius S et al.

Data associated with the article are available under the terms of the Creative Commons Zero "No rights reserved" data waiver (CC0 1.0 Public domain dedication). 

Dataset 1: Notes of observations from round 1 and round 2 panel meetings DOI: 10.5256/f1000research.12496.d178727 ( Gregorius *et al.*, 2017)

Transcriptions of interviews with panel members are available from the corresponding author on request. They have not been made available as a dataset because they cannot be de-identified without compromising anonymity and the ethical approval conditions for the project stated that only the research team would have access to the data.

## Supplementary material

Supplementary file 1: Observation matrixes Round 1

Supplementary file 2: Observation matrixes Round 2

Supplementary file 3: Interview guides for panel members for round 1 and round 2 awards

## References

[ref-1] Abdoul H Perrey C Amiel P: Peer review of grant applications: criteria used and qualitative study of reviewer practices. *PLoS One.* 2012;7(9):e46054. 10.1371/journal.pone.0046054 23029386 PMC3460995

[ref-2] Ahlquist V Andersson J Hahn Berg B: Observations on gender equality in a selection of The Swedish Research Council’s evaluation panels. Stockholm: Vetenskapsrådet.2013 Reference Source

[ref-26] Barnett AG Herbert DL Campbell M: Streamlined research funding using short proposals and accelerated peer review: an observational study. *BMC Health Serv Res.* 2015;15:55. 10.1186/s12913-015-0721-7 25888975 PMC4324047

[ref-3] Bates I Boyd A Smith H: A practical and systematic approach to organisational capacity strengthening for research in the health sector in Africa. *Health Res Policy Syst.* 2014;12:11. 10.1186/1478-4505-12-11 24581148 PMC3975897

[ref-4] Coryn CL Hattie JA Scriven M: Models and mechanisms for evaluating government-funded research: An international comparison. *Am J Eval.* 2007;28(4):437–457. 10.1177/1098214007308290

[ref-28] Dean L Gregorius S Bates I: Advancing the science of health research capacity strengthening in low-income and middle-income countries: a scoping review of the published literature, 2000–2016. *BMJ Open.* 2017;7(12):e018718. 10.1136/bmjopen-2017-018718 29217727 PMC5728300

[ref-5] Dean L Njelesani J Smith H: Promoting sustainable research partnerships: a mixed-method evaluation of a United Kingdom-Africa capacity strengthening award scheme. *Health Res Policy Syst.* 2015;13(1):81–90. 10.1186/s12961-015-0071-2 26695073 PMC4689047

[ref-25] Demicheli V Di Pietrantonj C: Peer review for improving the quality of grant applications. *Cochrane Database Syst Rev.* 2007; (2):MR000003. 10.1002/14651858.MR000003.pub2 17443627 PMC8973940

[ref-6] El Ansari W Maxwell AE Mikolajczyk RT: Promoting public health: benefits and challenges of a Europeanwide research consortium on student health. *Cent Eur J Public Health.* 2007;15(2):58–65. 17645218 10.21101/cejph.a3418

[ref-7] ESSENCE: Seven principles for strengthening research capacity in low- and middle-income countries: simple ideas in a complex world.2014; (retrieved 28.07.2017). Reference Source

[ref-8] Fogelholm M Leppinen S Auvinen A: Panel discussion does not improve reliability of peer review for medical research grant proposals. *J Clin Epidemiol.* 2012;65(1):47–52. 10.1016/j.jclinepi.2011.05.001 21831594

[ref-9] Gadsby EW: Research capacity strengthening: donor approaches to improving and assessing its impact in low- and middle-income countries. *Int J Health Plann Manage.* 2011;26(1):89–106. 10.1002/hpm.1031 20422620

[ref-10] Gregorius S Dean L Cole DC: Dataset 1 in: The peer review process for awarding funds to international science research consortia: a qualitative developmental evaluation. *F1000Research.* 2017 Data Source 10.12688/f1000research.12496.1 PMC5750705 29333239

[ref-29] Guthrie S Ghiga I Wooding S: What do we know about grant peer review in the health sciences? [version 1; referees: 1 approved, 1 approved with reservations]. *F1000Res.* 2017;6:1335 10.12688/f1000research.11917.1 PMC5883382 29707193

[ref-11] Jayasinghe UW Marsh HW Bond N: A new reader trial approach to peer review in funding research grants: An Australian experiment. *Scientometrics.* 2006;69(3):591–606. 10.1007/s11192-006-0171-4

[ref-12] Johnson VE: Statistical analysis of the National Institutes of Health peer review system. *Proc Natl Acad Sci U S A.* 2008;105(32):11076–11080. 10.1073/pnas.0804538105 18663221 PMC2488382

[ref-13] Klein T Olbrecht M: Triangulation of qualitative and quantitative methods in panel peer review research. *International Journal for Cross-Disciplinary Subjects in Education (IJCDSE).* 2011;2(2):342–348. Reference Source

[ref-14] Lamont M: How Professors Think: Inside the Curious World of Academic Judgement. Harvard University Press,2009 Reference Source

[ref-15] Lamont M Huutoniemi K: Comparing Customary Rules of Fairness: Evaluative practices in various types of peer review panels. In Camic C, Gross N and Lamont M (eds.) *Social knowledge in the making.*Chicago: University of Chicago Press,2012;209–232. Reference Source

[ref-16] Lawrenz F Thao M Johnson K: Expert panel reviews of research centers: the site visit process. *Eval Program Plann.* 2012;35(3):390–397. 10.1016/j.evalprogplan.2012.01.003 22306932

[ref-17] McNaughton Nicholls C Mills L Kotecha M: Observation. In Ritchie J, Lewis J, McNaughton Nicholls C & Ormston R (eds.) *Qualitative Research Practice: A Guide for Social Science Students and Researchers.*Second edition. London: Sage Publications,2013;243–268. Reference Source

[ref-27] Moore S Neylon C Paul Eve M: “Excellence R Us”: university research and the fetishisation of excellence. *Palgrave Communications.* 2017;3:16105 10.1057/palcomms.2016.105

[ref-18] Patton MQ: Developmental evaluation: Applying complexity concepts to enhance innovation and use. Guilford Press,2011 Reference Source

[ref-20] Research Council United Kingdom (RCUK): Report of the Research Councils UK Efficiency and Effectiveness of Peer Review Project.2006; (retrieved 28 June 2017). Reference Source

[ref-21] Syed SB Dadwal V Rutter P: Developed-developing country partnerships: benefits to developed countries? *Global Health.* 2012;8(1):17. 10.1186/1744-8603-8-17 22709651 PMC3459713

[ref-22] The United Kingdom Collaborative on Development Sciences (UKCDS): Health Research Capacity Strengthening: A UKCDS Mapping.2015; (Retrieved 28.07.2017). Reference Source

[ref-23] Wessely S: Peer review of grant applications: what do we know? *Lancet.* 1998;352(9124):301–305. 10.1016/S0140-6736(97)11129-1 9690424

[ref-24] Wooten KC Rose RM Ostir GV: Assessing and evaluating multidisciplinary translational teams: a mixed methods approach. *Eval Health Prof.* 2014;37(1):33–49. 10.1177/0163278713504433 24064432 PMC4180502

