# Supplementary material for: The peer review process for awarding funds to international science research consortia: a qualitative developmental evaluation
Source: F1000Res. 2018 Jan 16;6:1808. Originally published 2017 Oct 6. [Version 3] doi: 10.12688/f1000research.12496.3 (PMC5750705; doi:10.12688/f1000research.12496.3)
Supplement: Notes of observations from round 1 and round 2 panel meetings [file f1000research-6-14918-s0000.tgz › a7e74f83-fbe4-4a20-b569-fa06db40ef84_Dataset_1.docx]

Dataset files

*Dataset 1. Notes of observations from round 1 and round 2 panel meetings*

Notes of observations from round 1 panel meeting

| **Name** | **Content** | **Process** |
| --- | --- | --- |
| Observer 1 | - Focus on scientific excellence and innovation - Importance of capacity strengthening, relevance for Africa, risk and sustainability has been pointed out - Should PhD students be named? - Seems there is ambiguity among panel members regarding named PhD students - Potential role for CRU: help with management of capacity strengthening, encourage team to include more women in the project - Issues regarding career age - Some panel members included personal knowledge - Capacity strengthening seems to be entirely focused on training at the individual level - Chair: asked people to focus on capacity strengthening sustainability and not gender or Africa relevance - Importance of capacity strengthening discussed, not so much the process of it - Panel considered the standard of reviews to be very high | - Ranking not discussed as it may bias discussions - Questions are welcome - Scoring is ambiguous and difficult to relate to comments - Unclear process of bringing reviewers’ comments together - Suggestion about change in the application process: encourage un-funded applications to re-apply for Round 2 - Nominated PhD seemed a criteria for some panel members and not for others, but it was not a requirement in the guidelines - General issues with online application form |
| Observer 2 | - Focus on science excellence, capacity strengthening was often used when it was not possible to decipher between other applications - Need to consider the weighting given the development priorities and sustainability in line with the funding call - Capacity strengthening was understood in terms of training. Possibility that this may link to application form structure - Issues of career age - Strengths of institutions was referred to a few times - Minimal reflection of location of applicants - Sustainability aspects (beyond funding stop) not much discussed - Reputation of reviewers considered | - Limited reference to scoring - Suggestion: more structured process of how applications are presented and discussed - Variations in time given to applications that lead reviewer was not supporting of - Variations in panel member’s speaking time - Different number of reviewers for each application - All panel members were well informed about the content of the proposal and the background of the PIs |
| Observer 3 | - Strong focus on science excellence. Capacity strengthening discussed in more detail when decision was made about ‘maybe’ applications - Only two panel members from sub-Saharan institutions (1 Francophone) 🡪 recommendation for Round 2 to include additional panel members from sub-Saharan countries (including Francophone countries) - Use of own experience to make judgements 🡪 question of subjectivity ‘I trust they can do this’ - Development relevance and sustainability received relatively little attention in discussions - Relatively little discussion on gender, francophone background and experience in developing countries - Strong emphasis on background, experience and publication record | - More time spent on applications recommended for a grant - Different ways of discussing an application 🡪 makes it difficult to compare application and to follow observation matrix. Recommendation for clearer guidance on how to discuss applications - Some panel members spoke more than others - Little conflict between the panel members; discussions until consensus was reached - Conflict of interest has been addressed - Panel members were ‘equally’ included in discussions. Chair ensured that there were no remaining questions/comments after each application was discussed - Discussion about online application form |

Notes of observations from round 2 panel meeting

**Notes of observer 1**

- Short paper on peer-review process – time taken versus quality; need to focus more on getting good response rate from fewer reviewers, but still maintain high number (e.g. 7) per application.
- Many emphasised need to define/revise state-of-the-art and/or consult with existing initiatives in the region/project site
- All considered reviewers’ comments, especially criticism and compared between reviewers
- Almost all presenters gave overall score as part of feedback
- Dissemination of research mentioned as a positive feature – is it part of the marking criteria
- Relevance for Africa – bought up by reviewers as criterion
- ‘well-published’ mentioned in relation to PIs several times
- “Exciting innovation” seemed to be important factor in decision about quality versus practical, no innovation.
- “Lack of hypothesis” used as a criterion
- Following the chair’s instructions some panel members restricted comments to science but others included CS, which did influence the decision. 🡪 Needed guidance to be implemented consistently
- Want to have searchable and shorter proposals influenced by feedback from applicants
- More sub-headings to make sure methods details are included and front page summary

**Notes of observer 2**

- Chair: ‘no point to appoint just for capacity strengthening component’
- Idea of peer-review paper
- One panel member questioned the scoring and review system, as the scoring sometimes does not correspond to the reviewers comments. ‘Relying on numbers is not always appropriate’
- Panel members had possibility to contribute to each proposal (facilitated by the chair, who directly asked members with background in certain areas)
- Panel member: ‘this panel member is very well-qualified’
- Capacity strengthening was only discussed after short listing. This was not mentioned prior to the selection process
- Panel member pointed out that learning is not only north to south, but south to north.
- Academic writing seemed to be the most important capacity strengthening activity
- Capacity building was used more than the capacity strengthening
- Issues of gender and language were not much discussed
- After the award selection process the panel members had the chance to give feedback. Issues that were discussed related to the application forms. It was remarked that the document should be better searchable and the hypotheses and research methods should be stated clearly. The importance of feedback was also highlighted by some panel members
