# Supplementary material for: The peer review process for awarding funds to international science research consortia: a qualitative developmental evaluation
Source: F1000Res. 2018 Jan 16;6:1808. Originally published 2017 Oct 6. [Version 3] doi: 10.12688/f1000research.12496.3 (PMC5750705; doi:10.12688/f1000research.12496.3)
Supplement: Supplementary file 2 [file f1000research-6-14918-s0001.tgz › 19eb77fd-8b3f-4358-98c3-bba2a83628ac.pdf]

Supplementary file 1: Observation matrixes Round 1

Information for observer

Category	Includes	Researchers should note
**Selection process and assessment criteria**	Time spent on certain sections of the application (e.g. UK vs African project partner, qualifications vs project, science vs capacity strengthening elements)<br>Focus given to various applicants<br>Use of informal information in the selection process<br>How conflict of interest is dealt with<br>Discussion time per applicant<br>How policy and procedure influence the selection process<br>Value of scoring/emphasis of the triage process<br>Level of influence different panel members have on the selection process<br>Any changes in decision and what these are based on	Gender<br>Age<br>Ethnicity<br>First language of applicant<br>Professional level/experience of application<br>Where possible quantify observation (e.g. time speakers etc.)<br>Assessment criteria used
**Verbal communication and interactions**	Who speaks to who<br>Length of time given to each individual to speak<br>The way people speak/express their opinion<br>Volume of speech<br>Languages or dialects spoken<br>Tone of voice	Gender<br>Age<br>Ethnicity<br>Role on the panel<br>Profession of speaker<br>Dynamic of interaction<br>Where possible quantify observation (e.g. time speakers etc)
**Non-verbal communication and interactions**	Placement in the room (in meetings and in breaks)<br>Who interacts and who doesn't interact	Gender<br>Age<br>Ethnicity<br>Role on the panel<br>Profession of speaker<br>Dynamic of interaction

Matrix 1: Details of application

.

UK/SSA Applicant	Applicant Age, gender, nationality, first language	Profession, Qualification, Level in Career	Time spent and content on qualifications section of application	Time spent and content on project details section of application	Notes on content and any other observations

Matrix 2: Details of panel members

Application Number	Panel Member details (Age, Gender, nationality, first language)	Role on Panel, area of expertise	Time given to speak	Notes (see above guide for things to include here).
