# Supplementary material for: The peer review process for awarding funds to international science research consortia: a qualitative developmental evaluation
Source: F1000Res. 2018 Jan 16;6:1808. Originally published 2017 Oct 6. [Version 3] doi: 10.12688/f1000research.12496.3 (PMC5750705; doi:10.12688/f1000research.12496.3)
Supplement: Supplementary file 3 [file f1000research-6-14918-s0002.tgz › 93e1881e-283c-4525-96fc-1f35a7c40bf9.pdf]

Supplementary file 2: Observation matrixes Round 2

SCIENTIFIC STRENGTH

Assessment criteria	Observation			
Quality of research				
Strong individual research projects	African partner 1	African partner 2	African partner 3	
Strong research background of scientists	UK lead	African partner 1	African partner 2	African partner 3
Complementarity of scientists				
Institutions' strong track record of specific research area(s)	UK lead	African partner 1	African partner 2	African partner 3
Excellent quality of proposed PhD research projects	African partner 1	African partner 2	African partner 3	
Financial plan				
Other notes				

CAPACITY STRENGTHENING

Assessment criteria	Observation			
Capable institutions, that utilise programme for long-term development	UK lead	African partner 1	African partner 2	African partner 3
Wider training programme for other researchers and technicians	UK lead	African partner 1	African partner 2	African partner 3
Clearly research-active lead members	UK lead	African partner 1	African partner 2	African partner 3
Likelihood that support will lead to institutional change and long-term benefits for African partners				

ADDITIONAL ASSESSMENT CRITERIA

Assessment criteria	Observation			
Female lead members	UK lead	African partner 1	African partner 2	African partner 3
African early to mid-career scientists	African partner 1	African partner 2	African partner 3	
Multidisciplinary application				
Non-Anglophone researchers	African partner 1	African partner 2	African partner 3	
Reviewers-related observations				
Other observations				
