# Supplementary material for: The peer review process for awarding funds to international science research consortia: a qualitative developmental evaluation
Source: F1000Res. 2018 Jan 16;6:1808. Originally published 2017 Oct 6. [Version 3] doi: 10.12688/f1000research.12496.3 (PMC5750705; doi:10.12688/f1000research.12496.3)
Supplement: Supplementary file 4 [file f1000research-6-14918-s0003.tgz › dadf7d40-7fbb-486e-86c5-ee692f3e6a91.pdf]

Supplementary file 3: Interview guides for panel members for round 1 and round 2 awards

Interview Guide for Round 1

General Background and Demographics

- What is the role of the panel?
- What is your role within panel?
    - Do you have any additional roles?
- How long have you been in this (these) role(s)?
- What is your involvement with (name of award-making institution) or this initiative beyond being a member of the Panel?
- What is your area of specialisation?
- What is the highest level of academic qualification you have obtained?
- What is your first language?
- Can you speak any other languages fluently?
- How old are you?

The Panel

- **Can you tell me what you think of the Award Panel and how it functions?**
- **What are your views on how the panel works as a team?**

Grant Making Process

- **Can you describe your role in the application process?**
    - **PROMPT:**
        - What input do you have to application forms?
        - What input do you have to the format of the application process?
        - What worked well?
        - What could be improved?
        - What caused delays within the process?
- **Can you describe your role in the review process?**
    - **PROMPT:**
        - What is the format of the review process?
        - Were there any conflicts of interest for you or others within the review process? How is this dealt with? How often did this occur? How do you feel this impacted upon the selection process?
        - What worked well?
        - What could be improved?
        - What caused delays within the process?

- **Can you describe the selection and rejection process?**
    - **PROMPT:**
        - What is the format?
        - Were there any conflicts of interest for you or others within the review process? How is this dealt with? How often did this occur? How do you feel this impacted upon the selection process?
        - What worked well?
        - What could be improved?
        - What caused delays within the process?
- **Overall, how does the grant making process in this initiative compare to other Panels you sit on?**

Scientific Excellence

- **What do you understand by the term scientific excellence?**
- **Can you tell me about the consideration given to scientific excellence within the Award selection process?**

Research Capacity Strengthening

- **What do you understand by the term capacity strengthening?**
- **What do you think ideal research capacity looks like?**
- **Can you tell me about any examples of programmes you know of or have been involved with that have focused on research capacity strengthening?**
    - What worked at the international, national, institutional, individual level?
    - Why do you think these methods were effective?
    - What could have been improved about these approaches?
- **Can you tell me about the consideration given to capacity strengthening within the Award selection process?**
    - How was it prioritised?
    - In your opinion, was there a clear and common understanding of capacity strengthening amongst panel members?

Partnerships

As I am sure you are aware, the success of the research consortia in this Award scheme is linked to how effective the research partnerships are.

- **Can you tell me, in your experience, what makes a successful research partnership?**
    - **PROMPT:**
        - Communication, clear roles and responsibilities, previous relationships

- **During Award selection, what consideration was given to partners collaborative experience?**
    - **PROMPT:**
        - What consideration was given to the way partnerships were formed?

        - What importance was placed on prior collaborative experience?
        - For Award applicants who had previously been awarded network grants, how did this influence the way their applications were reviewed?

Grant Implementation

- **What is your involvement with the Award after the selection process is complete?**
    - What other involvement could you have that may be beneficial in ensuring the success of the Award?

Thank you very much for taking the time to speak to me today. Is there anything else that you feel is important to add about your experiences so far as a Panel Member?

Interview Guide for Round 2

General Background and Demographics

- What is the role of the panel?
- What is your role within panel?
    - Do you have any additional roles?
- How long have you been in this (these) role(s)?
- What is your involvement with (name of award-making institution) or this initiative beyond being a member of the Panel?
- What is your area of specialisation?
- What is the highest level of academic qualification you have obtained?
- What is your first language?
- Can you speak any other languages fluently?
- How old are you?

The Panel

- **Can you tell me what you think of the Award Panel and how it functions?**
- **What are your views on how the panel works as a team?**

Grant Making Process

- **Can you describe your role in the application process?**
    - **PROMPT:**
        - What input do you have to application forms?
        - What input do you have to the format of the application process?
        - What worked well?
        - What could be improved?
        - What caused delays within the process?
        - How did the application process different from the first Round?

- **Can you describe your role in the review process?**
    - **PROMPT:**
        - What is the format of the review process?
        - Were there any conflicts of interest for you or others within the review process? How is this dealt with? How often did this occur? How do you feel this impacted upon the selection process?
        - What worked well?
        - What could be improved?
        - What caused delays within the process?
        - How did the review process compare to Round 1?
- **Can you describe the selection and rejection process?**
    - **PROMPT:**
        - What is the format?
        - Were there any conflicts of interest for you or others within the review process? How is this dealt with? How often did this occur? How do you feel this impacted upon the selection process?
        - What worked well?
        - What could be improved?
        - What caused delays within the process?
        - How did the selection and rejection process compare to Round 1?
- **Can you describe how the revised assessment criteria were used?**
    - **PROMPT:**
        - Was there a common understanding about the use of the criteria?
        - How did the revised guidelines influence the selection and rejection process?
        - What worked well using the revised guidelines?
        - What could have been improved?

- **Overall, how does this award making process compare to Round 1?**
    - **PROMPT:**
        - What worked better and why?
        - What didn't work well and why?
- **Overall, how does the grant making process in this initiative compare to other Panels you sit on?**

Scientific Excellence

- **What do you understand by the term scientific excellence?**
- **Can you tell me about the consideration given to scientific excellence within the Award selection process and how it compared to Round 1?**

Research Capacity Strengthening

- **What do you understand by the term capacity strengthening?**
- **What do you think ideal research capacity looks like?**

- **Can you tell me about any examples of programmes you know of or have been involved with that have focused on research capacity strengthening?**
    - What worked at the international, national, institutional, individual level?
    - Why do you think these methods were effective?
    - What could have been improved about these approaches?

- **Can you tell me about the consideration given to capacity strengthening within the Award selection process and how it compared to Round 1?**
    - How was it prioritised?
    - In your opinion, was there a clear and common understanding of capacity strengthening amongst panel members?
    - What were the differences between Round 1 and 2?

Partnerships

As I am sure you are aware, the success of the research consortia in this Award scheme is linked to how effective the research partnerships are.

- **Can you tell me, in your experience, what makes a successful research partnership?**
    - **PROMPT:**
        - Communication, clear roles and responsibilities, previous relationships
- **During Award selection, what consideration was given to partners collaborative experience?**
    - **PROMPT:**
        - What consideration was given to the way partnerships were formed?
        - What importance was placed on prior collaborative experience?
        - For Award applicants who had previously been awarded network grants, how did this influence the way their applications were reviewed?

Grant Implementation

- **What is your involvement with the Award after the selection process is complete?**
    - What other involvement could you have that may be beneficial in ensuring the success of the Award?

Thank you very much for taking the time to speak to me today. Is there anything else that you feel is important to add about your experiences so far as a Panel Member?
