# Supplementary material for: The peer review process for awarding funds to international science research consortia: a qualitative developmental evaluation
Source: F1000Res. 2018 Jan 16;6:1808. Originally published 2017 Oct 6. [Version 3] doi: 10.12688/f1000research.12496.3 (PMC5750705; doi:10.12688/f1000research.12496.3)
Supplement: Peer review history

# Peer review: Referee response for version 2

**DOI:** 10.5256/f1000research.14698.r29265

**Published:** 2018-01-02

**Authors:** Adrian Barnett

**Affiliations:** 1 Institute of Health and Biomedical Innovation, Queensland University of Technology, Kelvin Grove, Qld, Australia

The authors have answered my queries well and I have only minor comments.

 The previous Cochrane review of funding peer review is probably also worth reading and citing: “Peer review for improving the quality of grant applications”, 18 April 2007 ^1^.

 The “success” of the panel (mentioned in the “Post award interviews with panel members” section) is the perceived success of how the applications were discussed and not whether the panel selected the best applications. Maybe this should be “successful functioning of the panel” or similar.

 Minor comment

 •    Double full stop at the end of the sentence, “Post award interviews with panel members…”

I have read this submission. I believe that I have an appropriate level of expertise to confirm that it is of an acceptable scientific standard.

## References

[1] DemicheliVDi PietrantonjC: Peer review for improving the quality of grant applications. *Cochrane Database Syst Rev*.2007; 10.1002/14651858.MR000003.pub2 MR000003 10.1002/14651858.MR000003.pub2 17443627PMC8973940

# Peer review: Referee response for version 1

**DOI:** 10.5256/f1000research.13530.r28056

**Published:** 2017-11-20

**Authors:** Adrian Barnett

**Affiliations:** 1 Institute of Health and Biomedical Innovation, Queensland University of Technology, Kelvin Grove, Qld, Australia

It’s good to read a thoughtful reflection on how peer review panels function given their importance for research. This study looked at a funding scheme that covered a range of fields and had an important aim of funding international groups to work on overseas aid projects.

 It would be worthwhile citing the recent systematic review in this area (Guthrie *et al., *2017 ^1^), which includes some additional references that consider the question whether panel meetings are worth the additional costs compared with independent reviews.

 The panel’s gender split was nowhere near 50:50. This was commented on in the results in that it: “proved difficult given the relative paucity of women and African scientists in these three research areas.” Part of the criteria for funding is “Support for female scientists”, but the group organizing the panel is ironically not adhering to this. Taking part in a panel can benefit researchers as it is a tremendous way to learn how to write a good proposal. As Guthrie *et al *state, “Members of funding panels may also benefit directly from their membership.” Hence not selecting women on the panel may just perpetuate the “paucity” of female scientists in this area and the funding agencies could be a key group for addressing this problem.

 Was there any discussion of the funding line and how many proposals might be funded? This often shapes the discussion as if only a handful of proposals can be funded then the panels members know that only the very best will get funding.

 The authors state: “There is evidence that pooling scores across a panel increases reliability compared to using scores produced by individuals’ reviewers (Fogelholm *et al.*, 2012)” But the main conclusion of Fogelholm’s paper was: “panel discussions per se did not improve the reliability of the evaluation.”

 What order were proposals discussed in? The order of the day can matter (e.g., reviewers being more alert in the morning), and ideally the proposals should be assessed in a random order to remove any long-term biases (e.g., from alphabetical ordering).

 What feedback was given to applicants? This is an important part of the process and given that panels cost time and money to assemble it would be worth giving detailed feedback so that applicants can improve. A funding scheme that I was involved in gave audio transcripts of the panel meeting to applicants (Barnett *et al., *2015) ^2^ , which was greatly appreciated by applicants.

 This point maybe outside the scope of the paper, but there is a great emphasis on “excellence” in this funding scheme, but “excellence” has little meaning in research as pointed out here: https://www.nature.com/articles/palcomms2016105 ^3^, and the authors of that paper conclude that “soundness and capacity-building” are far better criteria.

 Minor comments:

- Did any of the panel members also have applications being assessed?
- It would be useful to include a link to the funding scheme or a list of the winning proposals.
- I was surprised to read the Journal Impact Factors were used, given that they are a poor proxy the individual quality of papers.
- The authors state: “The peer review process is known to be influenced by the constitution of the panel with more deference between members of multi-disciplinary panels in which their expertise does not substantially overlap (Lamont & Huutoniemi, 2012), a finding which was confirmed by this study.” I did not read this as a major finding of this study and it was not highlighted in the results, nor was there any formal analysis that covered this. I think this needs expanding in the results to make such a strong statement in the conclusion.
- “There was also greater consensus among the panel regarding applications to be recommended or not recommended for an award” This finding was not shown in results.

I have read this submission. I believe that I have an appropriate level of expertise to confirm that it is of an acceptable scientific standard, however I have significant reservations, as outlined above.

## References

[1] GuthrieSGhigaIWoodingS: What do we know about grant peer review in the health sciences?. *F1000Research*.2017;6: 10.12688/f1000research.11917.1 10.12688/f1000research.11917.1 29707193PMC5883382

[2] BarnettAGHerbertDLCampbellMDalyNRobertsJAMudgeAGravesN: Streamlined research funding using short proposals and accelerated peer review: an observational study. *BMC Health Serv Res*.2015;15: 10.1186/s12913-015-0721-7 55 10.1186/s12913-015-0721-7 25888975PMC4324047

[3] MooreSNeylonCPaul EveMPaul O’DonnellDPattinsonD: “Excellence R Us”: university research and the fetishisation of excellence. *Palgrave Communications*.2017;3: 10.1057/palcomms.2016.105 10.1057/palcomms.2016.105

# Response

**Published:** 2017-12-18

**Authors:** imelda bates

**Affiliations:** Liverpool School of Tropical Medicine, UK

**Response to Adrian Barnett**, Institute of Health and Biomedical Innovation, Queensland University of Technology, Kelvin Grove, Qld, Australia 

 It’s good to read a thoughtful reflection on how peer review panels function given their importance for research. This study looked at a funding scheme that covered a range of fields and had an important aim of funding international groups to work on overseas aid projects.

 It would be worthwhile citing the recent systematic review in this area (Guthrie *et al., *2017 ^1^), which includes some additional references that consider the question whether panel meetings are worth the additional costs compared with independent reviews.

**Response:** We agreed that this is a very relevant review and we have included some of its findings in the introduction and discussion sections. We too were surprised by the lack of evidence about the effectiveness of peer-review for guiding funding decisions and also the lack of thoughtful critique about the pros and cons of the process compared to other ways of making funding decisions. Hopefully the Guthrie *et a*l paper may start to stimulate a discussion, and then action, about more cost-effective ways to reach funding decisions.

 The panel’s gender split was nowhere near 50:50. This was commented on in the results in that it: “proved difficult given the relative paucity of women and African scientists in these three research areas.” Part of the criteria for funding is “Support for female scientists”, but the group organizing the panel is ironically not adhering to this. Taking part in a panel can benefit researchers as it is a tremendous way to learn how to write a good proposal. As Guthrie *et al *state, “Members of funding panels may also benefit directly from their membership.” Hence not selecting women on the panel may just perpetuate the “paucity” of female scientists in this area and the funding agencies could be a key group for addressing this problem.

**Response:** We have expanded discussion of the gender make-up of the panel in the discussion, and the potential consequences for lack of diversity of perpetuating inequality, and we have also mentioned of the role of funders in helping to make this happen.

 Was there any discussion of the funding line and how many proposals might be funded? This often shapes the discussion as if only a handful of proposals can be funded then the panels members know that only the very best will get funding.

**Response:** The scheme was specifically designed and budgeted to fund 10 consortia – we have made this clear in the introduction text.

 The authors state: “There is evidence that pooling scores across a panel increases reliability compared to using scores produced by individuals’ reviewers (Fogelholm *et al.*, 2012)” But the main conclusion of Fogelholm’s paper was: “panel discussions per se did not improve the reliability of the evaluation.”

**Response:** We have altered the wording to more closely reflect the conclusion of this paper

 What order were proposals discussed in? The order of the day can matter (e.g., reviewers being more alert in the morning), and ideally the proposals should be assessed in a random order to remove any long-term biases (e.g., from alphabetical ordering).

**Response:** This is an important consideration which forgot to mention in the paper – the applications were not discussed in any particular order (e.g. alphabetically) and we have included a statement to explain this

 What feedback was given to applicants? This is an important part of the process and given that panels cost time and money to assemble it would be worth giving detailed feedback so that applicants can improve. A funding scheme that I was involved in gave audio transcripts of the panel meeting to applicants (Barnett *et al., *2015) ^2^ , which was greatly appreciated by applicants.

**Response:** This sounds like a great idea and would potentially be very helpful to applicants so we have stressed the need to improve the quality of feedback to applicants and mentioned audio transcripts in the discussion.

 This point maybe outside the scope of the paper, but there is a great emphasis on “excellence” in this funding scheme, but “excellence” has little meaning in research as pointed out here: https://www.nature.com/articles/palcomms2016105 ^3^, and the authors of that paper conclude that “soundness and capacity-building” are far better criteria.

**Response:** We too are not comfortable with the idea of ‘excellence’ but this was the term used in the panel discussions. We have quoted the Nature reference you suggested in an attempt to encourage readers to think critically about this term in the context of research

 Minor comments:

- Did any of the panel members also have applications being assessed? **Response:** there were a couple of occasions when members declared a conflict of interest because they had a connection with the applicants. They were not present in the room during discussions about these applications
- It would be useful to include a link to the funding scheme or a list of the winning proposals.
- **Response:** In our ethics application for this research we gave an undertaking to maintain anonymity for the particiaptns (i.e. members) so we are not able to provide details of the scheme
- I was surprised to read the Journal Impact Factors were used, given that they are a poor proxy the individual quality of papers.
- **Response:** We agree that impact factors are increasingly recognised as a poor proxy for quality but we have presented the observations as we noted them at the time.
- The authors state: “The peer review process is known to be influenced by the constitution of the panel with more deference between members of multi-disciplinary panels in which their expertise does not substantially overlap (Lamont & Huutoniemi, 2012), a finding which was confirmed by this study.” I did not read this as a major finding of this study and it was not highlighted in the results, nor was there any formal analysis that covered this. I think this needs expanding in the results to make such a strong statement in the conclusion.
- **Response:** The original statement we included was inferred from some of the comments made by panel members during the interviews. However we recognise that an investigation of how multi-disciplinarity affects the review process was not an objective of this study and that the evidence to support this statement is not strong, so on reflection we have decided to remove it from the revised version. 
- “There was also greater consensus among the panel regarding applications to be recommended or not recommended for an award” This finding was not shown in results.
- **Response:** We have expanded a couple of the sections to highlight findings that indicate greater consensus among panel members in round two. The relevant findings relate to a) less time needed to discuss each application and b) better consensus on a capacity strengthening focus.

- **Is the work clearly and accurately presented and does it cite the current literature?**Partly
- **Is the study design appropriate and is the work technically sound?**Yes
- **Are sufficient details of methods and analysis provided to allow replication by others?**Yes
- **If applicable, is the statistical analysis and its interpretation appropriate? **Not applicable
- **Are all the source data underlying the results available to ensure full reproducibility?**Partly
- **Are the conclusions drawn adequately supported by the results?**No
**References**
  1. Guthrie S, Ghiga I, Wooding S: What do we know about grant peer review in the health sciences?. *F1000Research*. 2017; **6**. Publisher Full Text
    2. Barnett AG, Herbert DL, Campbell M, Daly N, Roberts JA, Mudge A, Graves N: Streamlined research funding using short proposals and accelerated peer review: an observational study. *BMC Health Serv Res*. 2015; **15**: 55 PubMed Abstract | Publisher Full Text
    3. Moore S, Neylon C, Paul Eve M, Paul O’Donnell D, Pattinson D: “Excellence R Us”: university research and the fetishisation of excellence. *Palgrave Communications*. 2017; **3**. Publisher Full Text

# Peer review: Referee response for version 1

**DOI:** 10.5256/f1000research.13530.r26762

**Published:** 2017-11-03

**Authors:** Yaso Kunaratnam

**Affiliations:** 1 UK Collaborative on Development Sciences (UKCDS), London, UK

This is a timely, well-written and important look at the under-researched area of evaluating peer review for multi-national consortia. 

 The rise in spend on global development research has meant that complex consortia models are increasingly being used, and the findings will be useful for funders to think about how they could improve effectiveness and practice in this area. A synthesis on the key findings and lessons learned (what works well e.g. pooling scores/what does not work well) would be really useful.

 Setting up dual-purpose consortia to achieve scientific excellence and strengthen research capacity is a popular model, and I am not surprised to see tensions and conflicting views on which are more important. As the authors suggest, weighting would help assessment. I also think that research capacity experts could be on future peer review panels to address some of the gaps mentioned in levels of understanding on the topic.

 The article highlights that multi-national dual-purpose consortia is a complex model to assess – it was interesting to see the number of areas that peer reviewers take into consideration in table 1. I could see a useful resource emerging to help funders prioritise. I agree that assessment criteria should prioritise areas most closely linked with the goals of the programme – it is a challenge however when there are so many considerations of interest to funders e.g. interdisciplinarity, equitable partnerships, ethics, research uptake/impact, sustainability of networks, expertise within the consortia to deliver on the dual-purpose.

 The authors could suggest further research or work that is needed to improve peer review. It would be good to know how peer reviewers are sourced to help inform how to address gaps in the diversity of panels, and also look at the quality of assessment guidelines and how they could be improved, e.g. could they offer frameworks to assess quality that are evidence-based. Beyond peer review, a broader look at whether dual-purpose consortia are the right model for purpose or how they could be improved would be valuable.

 The methodology is sound, with and limitations explained and ethical considerations approved. Recent and relevant literature is referenced.

 Minor comments include:

- Intro para 4 - sustainable research networks mentioned as a goal but there is no reference later in terms of assessment.
- Table 1 could include overall total for scientific strengths, capacity and other areas.
- 1st para under Discussion – last sentence on Lamont & Huutoniemi, 2012, I did not quite understand.
- Links to the Swedish Research Council publication and statistical model would be useful, and funder assessment guidelines if they can be made accessible.

I have read this submission. I believe that I have an appropriate level of expertise to confirm that it is of an acceptable scientific standard.

# Response

**Published:** 2017-12-18

**Authors:** imelda bates

**Affiliations:** Liverpool School of Tropical Medicine, UK

**Response to Yaso Kunaratnam**,  UK Collaborative on Development Sciences (UKCDS), London, UK 

This is a timely, well-written and important look at the under-researched area of evaluating peer review for multi-national consortia. 

The rise in spend on global development research has meant that complex consortia models are increasingly being used, and the findings will be useful for funders to think about how they could improve effectiveness and practice in this area. A synthesis on the key findings and lessons learned (what works well e.g. pooling scores/what does not work well) would be really useful.

Setting up dual-purpose consortia to achieve scientific excellence and strengthen research capacity is a popular model, and I am not surprised to see tensions and conflicting views on which are more important. As the authors suggest, weighting would help assessment. I also think that research capacity experts could be on future peer review panels to address some of the gaps mentioned in levels of understanding on the topic.

**Response:** We have now mentioned the need to include research capacity experts on future peer review panels

The article highlights that multi-national dual-purpose consortia is a complex model to assess – it was interesting to see the number of areas that peer reviewers take into consideration in table 1. I could see a useful resource emerging to help funders prioritise. I agree that assessment criteria should prioritise areas most closely linked with the goals of the programme – it is a challenge however when there are so many considerations of interest to funders e.g. interdisciplinarity, equitable partnerships, ethics, research uptake/impact, sustainability of networks, expertise within the consortia to deliver on the dual-purpose.

**Response:** We have indicated that, if confirmed by further studies, the approach used in table 1 might be useful to help funders prioritise

The authors could suggest further research or work that is needed to improve peer review. It would be good to know how peer reviewers are sourced to help inform how to address gaps in the diversity of panels, and also look at the quality of assessment guidelines and how they could be improved, e.g. could they offer frameworks to assess quality that are evidence-based. Beyond peer review, a broader look at whether dual-purpose consortia are the right model for purpose or how they could be improved would be valuable.

**Response:** It is clear that further work is needed to understand more about the peer review process such as how it compares to other models, and whether consortia are the right models. Although a detailed discussion about these topics is beyond the scope of this paper we have included a couple of references that deal specifically with evidence and research on this topic including a very recent systematic review (Guthrie, et al 2017; Barnett, et al 2015)

The methodology is sound, with and limitations explained and ethical considerations approved. Recent and relevant literature is referenced.

Minor comments include:

- Intro para 4 - sustainable research networks mentioned as a goal but there is no reference later in terms of assessment.
- **Response:** Networks were not discussed by the panel members and we have stated this in the revised version.
- Table 1 could include overall total for scientific strengths, capacity and other areas.
- **Response:** Table 1 has been amended
- 1st para under Discussion – last sentence on Lamont & Huutoniemi, 2012, I did not quite understand.
- **Response:** This sentence has been adjusted
- Links to the Swedish Research Council publication and statistical model would be useful, and funder assessment guidelines if they can be made accessible
- **Response:** Details of the assessment process including sections on criteria, openness and gender equality, are available on the Swedish research Council’s website at https://www.vr.se/inenglish/researchfunding/assessment/assessmentcriteria.4.7257118313b2995b0f27ace.html (accessed 15 December 2017) 
